# Supplementary material for: Cross-Platform Transcriptomic Data Integration, Profiling, and Mining in Vibrio cholerae
Source: Microbiol Spectr. 2023 May 16;11(3):e05369-22. doi: 10.1128/spectrum.05369-22 (PMC10269641; doi:10.1128/spectrum.05369-22)
Supplement: Supplemental file 1 — Supplemental material. Download spectrum.05369-22-s0001.pdf, PDF file, 3.3 MB [file spectrum.05369-22-s0001.pdf]

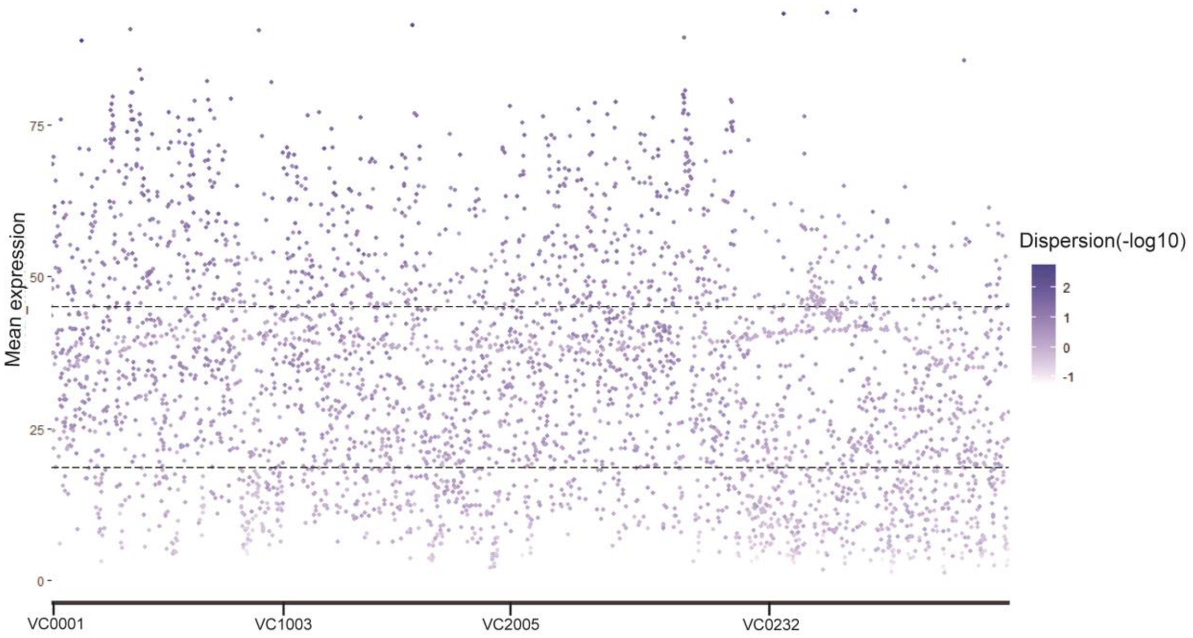

Figure S1 Gene mean expression and dispersion distribution of all genes

The horizontal coordinate is the gene of *V. cholerae*, the vertical coordinate is the corresponding mean expression of each gene, and the dashed line is the quartile of the mean expression of all genes, 45.13 for the upper dashed line and 18.57 for the lower dashed line. the color of each point represents the magnitude of dispersion, and the numerical and color correspondence is shown on the right.

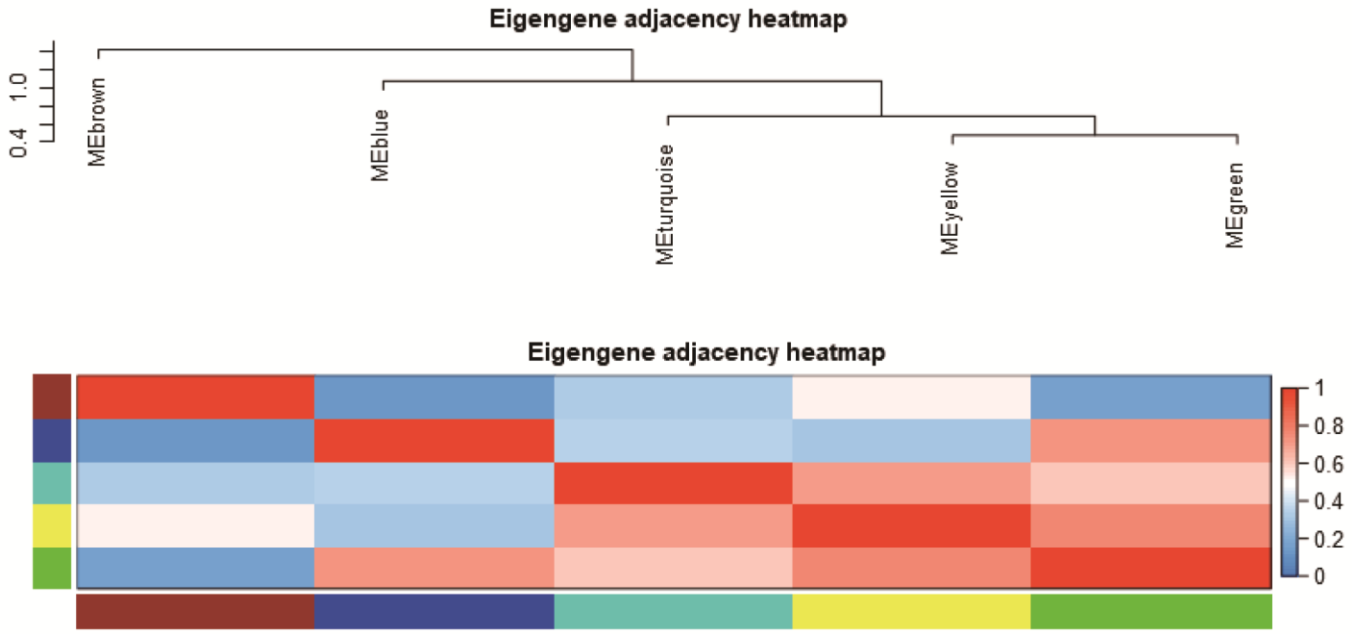

Figure S2 Correlation between different functional modules

The length of the vertical lines reflects the correlation between the modules (short lines are indicative of greater correlation). And the color of the square grids also reflects the correlation between the modules (red grids are indicative of greater correlation).

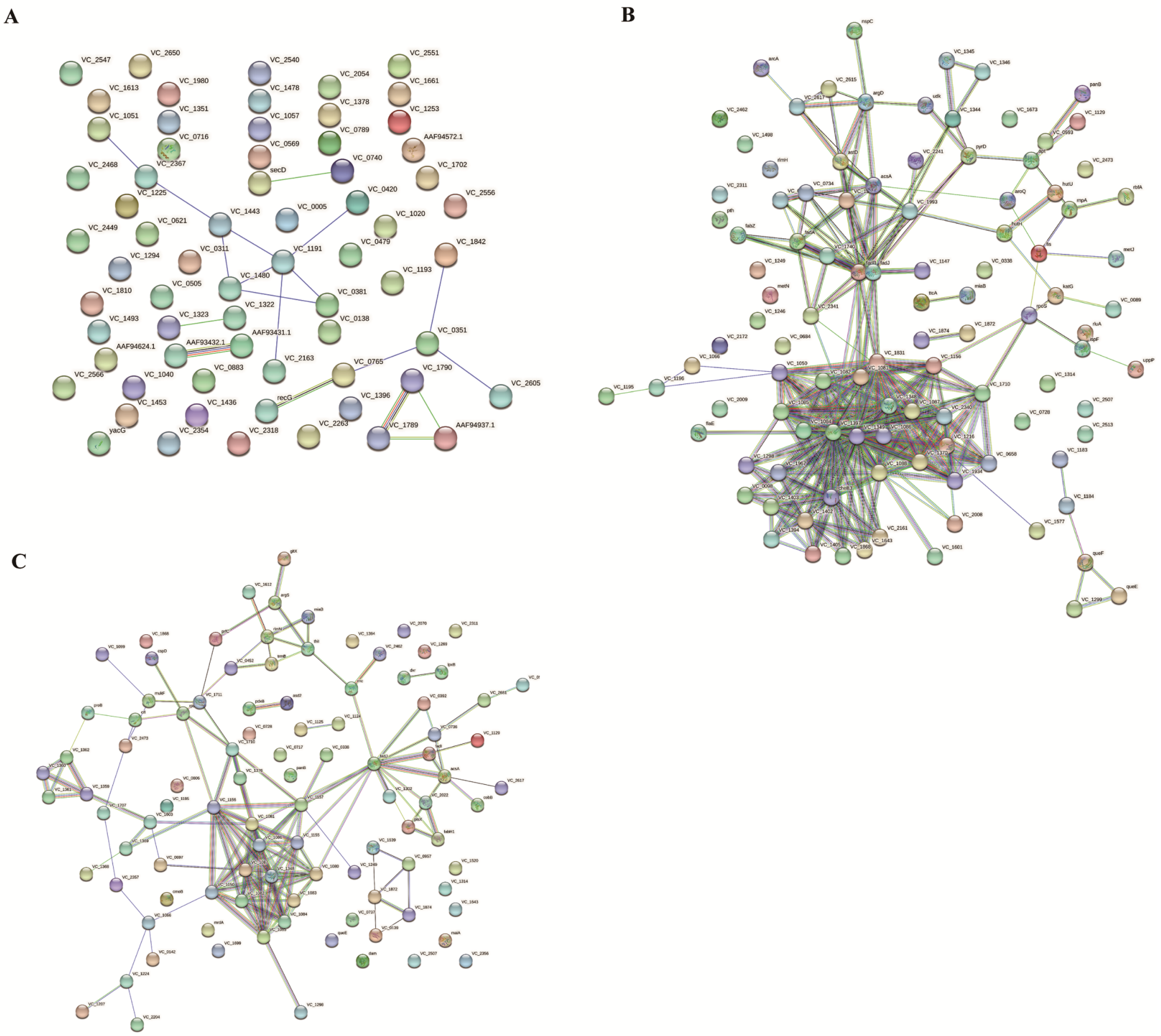

Figure S3 PPI interaction networks between hub genes from STRING database

(A) PPI network diagram of hub genes within the turquoise module. (B) PPI network diagram of hub genes within the blue module. (C) PPI network diagram of hub genes within the brown module.

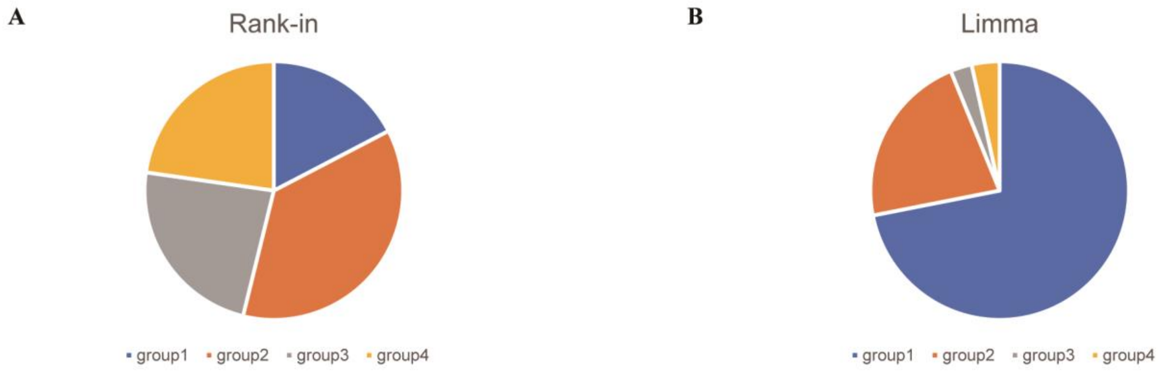

Figure S4 Sample clustering by Rank-in and the Limma R package normalizedBetweenArrays function

(A) Sample clustering proportion of Rank-in. (B) Sample clustering proportion of the Limma R package normalizedBetweenArrays function.

A

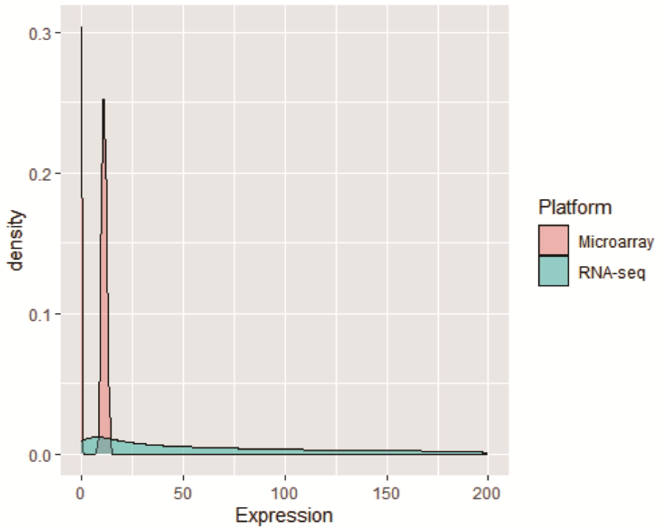

Figure S5 Expression distribution density plot of raw data

The blue part is the expression distribution density plot of RNA-seq data, and the pink part is the expression distribution density plot of microarray data.

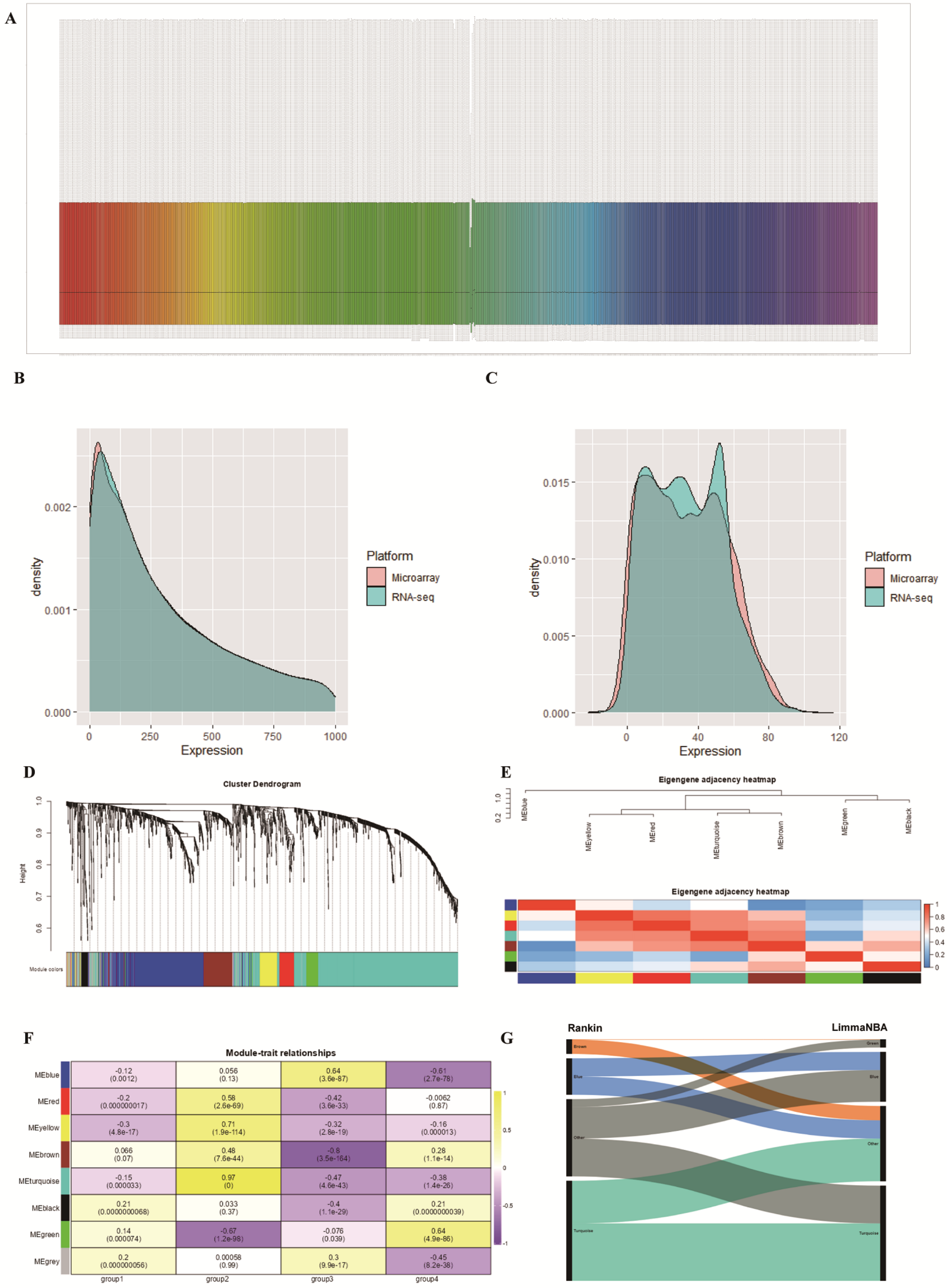
A
B
0.002
0.001
0.000
density
0
250
500
750
1000
Expression
Platform
Microarray
RNA-seq
C
0.015
0.010
0.005
0.000
density
0
40
80
120
Expression
Platform
Microarray
RNA-seq
D
Cluster Dendrogram
Height
Module colors
E
Eigengene adjacency heatmap
MEblue
MEyellow
MEred
MEturquoise
MEbrown
MEgreen
MEblack
Eigengene adjacency heatmap
F
Module-trait relationships
MEblue -0.12 (0.0012) 0.056 (0.13) 0.64 (3.6e-87) -0.61 (2.7e-78)
MEred -0.2 (0.000000017) 0.58 (2.6e-69) -0.42 (3.6e-33) -0.0062 (0.87)
MEyellow -0.3 (4.8e-17) 0.71 (1.9e-114) -0.32 (2.8e-19) -0.16 (0.000013)
MEbrown 0.066 (0.07) 0.48 (7.6e-44) -0.8 (3.5e-164) 0.28 (1.1e-14)
MEturquoise -0.15 (0.000033) 0.97 (0) -0.47 (4.6e-43) -0.38 (1.4e-26)
MEblack 0.21 (0.0000000068) 0.033 (0.37) -0.4 (1.1e-29) 0.21 (0.0000000039)
MEgreen 0.14 (0.000074) -0.67 (1.2e-98) -0.076 (0.039) 0.64 (4.9e-86)
MEgrey 0.2 (0.000000056) 0.00058 (0.99) 0.3 (9.9e-17) -0.45 (8.2e-38)
group1
group2
group3
group4
G
Rankin
LimmaNBA

Figure S6 The Limma R package normalizedBetweenArrays function demonstrates similar results

(A) Transcripts of the whole sample after integration (horizontal coordinates are corrected gene expression values). (B) Expression distribution density plot of the Limma R package normalizedBetweenArrays function data. (C) Expression distribution density plot of the Rank-in data. (D) Cluster dendrogram of different genes identified by WGCNA assigned to different modules. (E) Correlation between different functional modules. (F) Correlation between modules and groups (The upper values in the boxes are correlation coefficients and the lower values are *p*-values.). (G) Sankey diagrams of genes within the modules of the two integration methods.

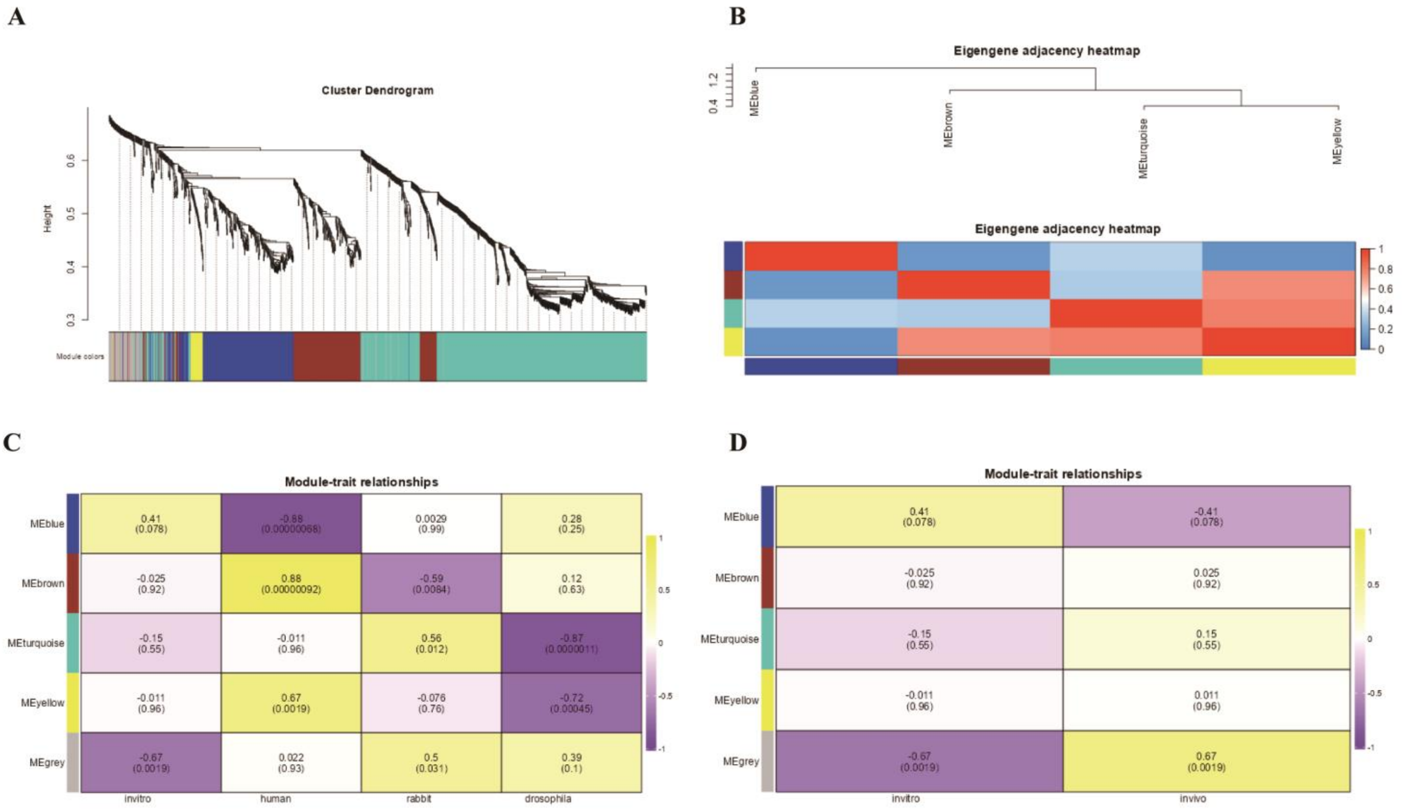

Figure S7 Inconsistent *V. cholerae* gene expression *in vivo* between hosts

(A) Transcripts of the whole sample after integration (horizontal coordinates are corrected gene expression values). (B) Cluster dendrogram of different genes identified by WGCNA assigned to different modules. (C) Correlation between modules and groups using different hosts (The upper values in the boxes are correlation coefficients and the lower values are *p*-values.). (D) Correlation between modules and groups using *in vitro* or *in vivo* (The upper values in the boxes are correlation coefficients and the lower values are *p*-values.).
