# Supplementary material for: Cross-Platform Transcriptomic Data Integration, Profiling, and Mining in Vibrio cholerae
Source: Microbiol Spectr. 2023 May 16;11(3):e05369-22. doi: 10.1128/spectrum.05369-22 (PMC10269641; doi:10.1128/spectrum.05369-22)
Supplement: Supplemental file 2 — Supplemental material. Download spectrum.05369-22-s0002.pdf, PDF file, 0.2 MB [file spectrum.05369-22-s0002.pdf]

Sample ID	Project ID
ERR5101704	PRJEB42488
ERR5101705	PRJEB42488
ERR5101706	PRJEB42488
ERR5101707	PRJEB42488
ERR5101708	PRJEB42488
ERR5101709	PRJEB42488
ERR5101710	PRJEB42488
ERR5101711	PRJEB42488
ERR5101712	PRJEB42488
ERR5101713	PRJEB42488
ERR5101714	PRJEB42488
SRR10387915	PRJNA587223
SRR10387916	PRJNA587223
SRR10387917	PRJNA587223
SRR10387918	PRJNA587223
SRR10387921	PRJNA587223
SRR10387922	PRJNA587223
SRR10480743	GSE140515
SRR10480744	GSE140516
SRR10480745	GSE140517
SRR10480746	GSE140518
SRR10480747	GSE140519
SRR10480748	GSE140520
SRR10905341	PRJNA601792
SRR10905342	PRJNA601792
SRR10905343	PRJNA601792
SRR10905344	PRJNA601792
SRR10905351	PRJNA601792
SRR10905362	PRJNA601792
SRR10905369	PRJNA601792
SRR10905370	PRJNA601792
SRR10905371	PRJNA601792
SRR10905372	PRJNA601792
SRR10905373	PRJNA601792
SRR10905376	PRJNA601792
SRR10905377	PRJNA601792
SRR10905378	PRJNA601792
SRR10905379	PRJNA601792
SRR10905380	PRJNA601792
SRR10905381	PRJNA601792
SRR10905382	PRJNA601792
SRR10905383	PRJNA601792
SRR10905384	PRJNA601792
SRR10905385	PRJNA601792
SRR10905386	PRJNA601792
SRR10905387	PRJNA601792
SRR10905388	PRJNA601792
SRR10905389	PRJNA601792
SRR10905390	PRJNA601792
SRR10905391	PRJNA601792
SRR10905392	PRJNA601792
SRR10905393	PRJNA601792
SRR10905394	PRJNA601792
SRR10905395	PRJNA601792
SRR10905396	PRJNA601792
SRR10985166	GSE144478
SRR10985167	GSE144478

SRR10985168	GSE144478
SRR10985169	GSE144478
SRR10985170	GSE144478
SRR10985171	GSE144478
SRR10985172	GSE144478
SRR10985173	GSE144478
SRR10985174	GSE144478
SRR10985175	GSE144478
SRR10985176	GSE144478
SRR10985177	GSE144478
SRR10985178	PRJNA603761
SRR10985179	PRJNA603761
SRR10985180	PRJNA603761
SRR10985181	PRJNA603761
SRR10985182	PRJNA603761
SRR10985183	PRJNA603761
SRR11091459	PRJNA606590
SRR11091460	PRJNA606590
SRR11091461	PRJNA606590
SRR11091462	PRJNA606590
SRR11091463	PRJNA606590
SRR11091464	PRJNA606590
SRR11148747	GSE145764
SRR11148748	GSE145764
SRR11148749	GSE145764
SRR11148750	GSE145764
SRR11148751	GSE145764
SRR11148752	GSE145764
SRR11194666	PRJNA609114
SRR11194667	PRJNA609114
SRR11194668	PRJNA609114
SRR11194669	PRJNA609114
SRR11194670	PRJNA609114
SRR11194671	PRJNA609114
SRR11194672	PRJNA609114
SRR11194673	PRJNA609114
SRR11194674	PRJNA609114
SRR11194675	PRJNA609114
SRR11194676	PRJNA609114
SRR11194677	PRJNA609114
SRR11194678	PRJNA609114
SRR11194679	PRJNA609114
SRR11194680	PRJNA609114
SRR11194681	PRJNA609114
SRR11194682	PRJNA609114
SRR11194683	PRJNA609114
SRR11194684	PRJNA609114
SRR11194685	PRJNA609114
SRR11194686	PRJNA609114
SRR11194687	PRJNA609114
SRR11194688	PRJNA609114
SRR11194689	PRJNA609114
SRR11194690	PRJNA609114
SRR11194691	PRJNA609114
SRR11194692	PRJNA609114
SRR11194693	PRJNA609114
SRR11194694	PRJNA609114
SRR11194695	PRJNA609114

SRR11194696	PRJNA609114
SRR11194697	PRJNA609114
SRR11194698	PRJNA609114
SRR11194699	PRJNA609114
SRR11194700	PRJNA609114
SRR11194701	PRJNA609114
SRR11194702	PRJNA609114
SRR11194703	PRJNA609114
SRR11194704	PRJNA609114
SRR11194705	PRJNA609114
SRR11194706	PRJNA609114
SRR11194707	PRJNA609114
SRR11194708	PRJNA609114
SRR11194709	PRJNA609114
SRR11194710	PRJNA609114
SRR11194711	PRJNA609114
SRR11194712	PRJNA609114
SRR11194713	PRJNA609114
SRR11194714	PRJNA609114
SRR11194715	PRJNA609114
SRR11194716	PRJNA609114
SRR11194717	PRJNA609114
SRR11194718	PRJNA609114
SRR11194719	PRJNA609114
SRR11194720	PRJNA609114
SRR11194721	PRJNA609114
SRR11194722	PRJNA609114
SRR11194723	PRJNA609114
SRR11194724	PRJNA609114
SRR11194725	PRJNA609114
SRR11194726	PRJNA609114
SRR11194727	PRJNA609114
SRR11194728	PRJNA609114
SRR11194729	PRJNA609114
SRR11194730	PRJNA609114
SRR11194731	PRJNA609114
SRR11194732	PRJNA609114
SRR11194733	PRJNA609114
SRR11194734	PRJNA609114
SRR11194735	PRJNA609114
SRR11546813	GSE148675
SRR11546814	GSE148675
SRR11546815	GSE148675
SRR11546816	GSE148675
SRR11546817	GSE148675
SRR11546818	GSE148675
SRR11546819	GSE148675
SRR11546820	GSE148675
SRR11546821	GSE148675
SRR11546822	GSE148675
SRR11546823	GSE148675
SRR11546824	GSE148675
SRR12867119	PRJNA670462
SRR12867120	PRJNA670462
SRR12867121	PRJNA670462
SRR12867122	PRJNA670462
SRR12867123	PRJNA670462
SRR12867124	PRJNA670462

SRR12867125	PRJNA670462
SRR12867126	PRJNA670462
SRR12867127	PRJNA670462
SRR12867128	PRJNA670462
SRR12867129	PRJNA670462
SRR12867130	PRJNA670462
SRR12867131	PRJNA670462
SRR12867132	PRJNA670462
SRR12867133	PRJNA670462
SRR12867134	PRJNA670462
SRR12867135	PRJNA670462
SRR12867136	PRJNA670462
SRR13362016	GSE164298
SRR13362017	GSE164298
SRR13362018	GSE164298
SRR13362019	GSE164298
SRR13362020	GSE164298
SRR13362021	GSE164298
SRR13362022	GSE164298
SRR13362023	GSE164298
SRR1382192	PRJNA252504
SRR1382205	PRJNA252504
SRR1382206	PRJNA252504
SRR1382207	PRJNA252504
SRR1382208	PRJNA252504
SRR1382209	PRJNA252504
SRR1382210	PRJNA252504
SRR1382211	PRJNA252504
SRR1521326	PRJNA255655
SRR1521327	PRJNA255655
SRR1521328	PRJNA255655
SRR1521329	PRJNA255655
SRR1521330	PRJNA255655
SRR1521331	PRJNA255655
SRR1521332	PRJNA255655
SRR1521333	PRJNA255655
SRR1521334	PRJNA255655
SRR1521335	PRJNA255655
SRR1521336	PRJNA255655
SRR1521337	PRJNA255655
SRR1544143	PRJNA258016
SRR1544479	PRJNA258016
SRR1583199	PRJNA261682
SRR1583202	PRJNA261682
SRR1602499	GSE62084
SRR1602500	GSE62084
SRR1602501	GSE62084
SRR1602502	GSE62084
SRR1602503	GSE62084
SRR1602504	GSE62084
SRR1602505	GSE62084
SRR1602506	GSE62084
SRR1602507	GSE62084
SRR1602508	GSE62084
SRR1602509	GSE62084
SRR1602510	GSE62084
SRR1602511	GSE62084
SRR1602512	GSE62084

SRR1602513	GSE62084
SRR1602514	GSE62084
SRR1636723	GSE62785
SRR1636724	GSE62785
SRR1636725	GSE62785
SRR1636726	GSE62785
SRR1636727	GSE62785
SRR1636728	GSE62785
SRR1636729	GSE62785
SRR1636730	GSE62785
SRR1653344	PRJNA62185
SRR1653345	PRJNA62185
SRR1653346	PRJNA62185
SRR1653347	PRJNA62185
SRR1653348	PRJNA62185
SRR1653349	PRJNA62185
SRR2230414	PRJNA294584
SRR2244514	SRR2244514
SRR2244515	SRR2244515
SRR2302168	PRJNA295073
SRR2302169	PRJNA295073
SRR2302170	PRJNA295073
SRR2302171	PRJNA295073
SRR2302172	PRJNA295073
SRR2302173	PRJNA295073
SRR2302174	PRJNA295073
SRR2302175	PRJNA295073
SRR2302176	PRJNA295073
SRR2319527	PRJNA294584
SRR2319528	PRJNA294584
SRR2319529	PRJNA294584
SRR2319530	PRJNA294584
SRR2319531	PRJNA294584
SRR2319532	PRJNA294584
SRR2319533	PRJNA294584
SRR2319534	PRJNA294584
SRR2319535	PRJNA294584
SRR2648176	GSE73975
SRR2648177	GSE73975
SRR2648178	GSE73975
SRR2648179	GSE73975
SRR2648180	GSE73975
SRR2648181	GSE73975
SRR2648182	GSE73975
SRR2648183	GSE73975
SRR3274035	GSE79467
SRR3274036	GSE79467
SRR3274037	GSE79467
SRR3274038	GSE79467
SRR3274039	GSE79467
SRR3274040	GSE79467
SRR3331917	GSE79911
SRR3331918	GSE79911
SRR3331919	GSE79911
SRR3331920	GSE79911
SRR3331921	GSE79911
SRR3331922	GSE79911
SRR3331923	GSE79911

SRR3331924	GSE79911
SRR3331925	GSE79911
SRR3331926	GSE79911
SRR3331927	GSE79911
SRR3331928	GSE79911
SRR3331929	GSE79911
SRR3331930	GSE79911
SRR3371159	GSE80217
SRR3371160	GSE80217
SRR3371161	GSE80217
SRR3371162	GSE80217
SRR3371163	GSE80217
SRR3371164	GSE80217
SRR3371165	GSE80217
SRR3371166	GSE80217
SRR3371167	GSE80217
SRR3371168	GSE80217
SRR3371169	GSE80217
SRR3371170	GSE80217
SRR3586848	PRJNA314347
SRR3586850	PRJNA314347
SRR3586851	PRJNA314347
SRR3586853	PRJNA314347
SRR3586855	PRJNA314347
SRR3586857	PRJNA314347
SRR3586860	PRJNA314347
SRR3586863	PRJNA314347
SRR3586871	PRJNA314347
SRR3586876	PRJNA314347
SRR3586879	PRJNA314347
SRR3586886	PRJNA314347
SRR403875	PRJNA62185
SRR403876	PRJNA62185
SRR403877	PRJNA62185
SRR403878	PRJNA62185
SRR404005	PRJNA62185
SRR442059	GSE36373
SRR442060	GSE36373
SRR442061	GSE36373
SRR442062	GSE36373
SRR442063	GSE36373
SRR442064	GSE36373
SRR5519749	PRJNA374569
SRR5519750	PRJNA374569
SRR5519751	PRJNA374569
SRR5519752	PRJNA374569
SRR5519753	PRJNA374569
SRR5519754	PRJNA374569
SRR5688346	PRJNA390276
SRR5688347	PRJNA390276
SRR5688348	PRJNA390276
SRR5688349	PRJNA390276
SRR5688350	PRJNA390276
SRR5688351	PRJNA390276
SRR5938168	PRJNA314347
SRR5938199	PRJNA314347
SRR5938200	PRJNA314347
SRR5938201	PRJNA314347

SRR5938202	PRJNA314347
SRR5938203	PRJNA314347
SRR5938204	PRJNA314347
SRR5938256	PRJNA314347
SRR5938304	PRJNA314347
SRR5938305	PRJNA314347
SRR5938306	PRJNA314347
SRR5938307	PRJNA314347
SRR6334011	GSE107538
SRR6334012	GSE107538
SRR6334013	GSE107538
SRR6334014	GSE107538
SRR6334015	GSE107538
SRR6334016	GSE107538
SRR6334017	GSE107538
SRR6334018	GSE107538
SRR6334019	GSE107538
SRR6334020	GSE107538
SRR6334021	GSE107538
SRR6334022	GSE107538
SRR6334023	GSE107538
SRR6334024	GSE107538
SRR6334025	GSE107538
SRR6987751	PRJNA429784
SRR6987752	PRJNA429784
SRR6987753	PRJNA429784
SRR6987754	PRJNA429784
SRR6987755	PRJNA429784
SRR6987756	PRJNA429784
SRR6987757	PRJNA429784
SRR6987758	PRJNA429784
SRR6987759	PRJNA429784
SRR6987760	PRJNA429784
SRR6987761	PRJNA429784
SRR6987762	PRJNA429784
SRR6987763	PRJNA429784
SRR6987764	PRJNA429784
SRR6987765	PRJNA429784
SRR6987766	PRJNA429784
SRR6987767	PRJNA429784
SRR6987768	PRJNA429784
SRR6987769	PRJNA429784
SRR6987770	PRJNA429784
SRR6987771	PRJNA429784
SRR6987772	PRJNA429784
SRR6987773	PRJNA429784
SRR6987774	PRJNA429784
SRR7178725	GSE114592
SRR7178726	GSE114592
SRR7178727	GSE114592
SRR7178728	GSE114592
SRR7178729	GSE114592
SRR7178730	GSE114592
SRR7178731	GSE114592
SRR7178732	GSE114592
SRR7178733	GSE114592
SRR7178734	GSE114592
SRR7178735	GSE114592

SRR7178736	GSE114592
SRR7178737	GSE114592
SRR7178738	GSE114592
SRR7178739	GSE114592
SRR7178740	GSE114592
SRR7178741	GSE114592
SRR7178742	GSE114592
SRR7178743	GSE114592
SRR7178744	GSE114592
SRR7178745	GSE114592
SRR7178746	GSE114592
SRR7178747	GSE114592
SRR7178748	GSE114592
SRR7298325	GSE115711
SRR7298326	GSE115711
SRR7298327	GSE115711
SRR7298328	GSE115711
SRR7298329	GSE115711
SRR7298330	GSE115711
SRR7298331	GSE115711
SRR7298332	GSE115711
SRR7298333	GSE115711
SRR7298334	GSE115711
SRR7298335	GSE115711
SRR7298336	GSE115711
SRR7298337	GSE115711
SRR7298338	GSE115711
SRR7298339	GSE115711
SRR7298340	GSE115711
SRR7298341	GSE115711
SRR7298342	GSE115711
SRR7298343	GSE115711
SRR7298344	GSE115711
SRR7298345	GSE115711
SRR7298346	GSE115711
SRR7298347	GSE115711
SRR7298348	GSE115711
SRR7298349	GSE115715
SRR7298350	GSE115715
SRR7298351	GSE115715
SRR7298352	GSE115715
SRR7298353	GSE115715
SRR7298354	GSE115715
SRR8284470	PRJNA508600
SRR8284471	PRJNA508600
SRR8284472	PRJNA508600
SRR8284473	PRJNA508600
SRR8284474	PRJNA508600
SRR8284475	PRJNA508600
SRR8294795	PRJNA509145
SRR8294796	PRJNA509145
SRR8294797	PRJNA509145
SRR8294798	PRJNA509145
SRR8294799	PRJNA509145
SRR8294800	PRJNA509145
SRR8316512	PRJNA509993
SRR8316513	PRJNA509993
SRR8316514	PRJNA509993

SRR8316515	PRJNA509993
SRR8316516	PRJNA509993
SRR8316517	PRJNA509993
SRR8316518	PRJNA509993
SRR8316519	PRJNA509993
SRR8316520	PRJNA509993
SRR8316521	PRJNA509993
SRR8316522	PRJNA509993
SRR8316523	PRJNA509993
SRR8316524	PRJNA509993
SRR8316525	PRJNA509993
SRR8316526	PRJNA509993
SRR8316527	PRJNA509993
SRR8316528	PRJNA509993
SRR8316529	PRJNA509993
SRR8316530	PRJNA509993
SRR8316531	PRJNA509993
SRR8447493	GSE125160
SRR8447494	GSE125160
SRR8447495	GSE125160
SRR8447496	GSE125160
SRR8447497	GSE125160
SRR8447498	GSE125160
SRR8447499	GSE125160
SRR8447500	GSE125160
SRR8447501	GSE125160
SRR9289621	GSE132653
SRR9289622	GSE132653
SRR9289623	GSE132653
SRR9289624	GSE132653
SRR9289625	GSE132653
SRR9289626	GSE132653
SRR9289627	GSE132653
SRR9289628	GSE132653
SRR9313019	GSE132870
SRR9313020	GSE132870
SRR9313021	GSE132870
SRR9853383	GSE135010
SRR9853384	GSE135010
SRR9853385	GSE135010
SRR9853386	GSE135010
SRR9853387	GSE135010
SRR9853388	GSE135010
SRR9975249	GSE135887
SRR9975250	GSE135887
SRR9975251	GSE135887
SRR9975252	GSE135887
SRR9975253	GSE135887
GSM109602$ mid log LB 2 ug RNA aerobic	GSE4877
GSM109602$mid log M9 + 0.5% lactate aerobic (2 ug RNA)	GSE4877
GSM109603$ mid log LB 2 ug RNA aerobic	GSE4877
GSM109603$ mid log M9 + 0.5% lactate aerobic (2 ug RNA)	GSE4877

GSM109604$ mid log LB 2 ug RNA aerobic	GSE4877
GSM109604$ mid log M9 + 0.5% lactate aerobic (2 ug RNA)	GSE4877
GSM109605$ mid log LB 2 ug RNA aerobic	GSE4877
GSM109605$ mid log M9 + 0.5% lactate aerobic (2 ug RNA)	GSE4877
GSM109606$ mid log LB 2 ug RNA aerobic	GSE4877
GSM109606$ mid log M9 + 0.5% maltose aerobic (2 ug RNA)	GSE4877
GSM109607$ mid log LB 2 ug RNA aerobic	GSE4877
GSM109607$ mid log M9 + 0.5% maltose aerobic (2 ug RNA)	GSE4877
GSM109608$ mid log LB 2 ug RNA aerobic	GSE4877
GSM109608$ mid log M9 + 0.5% maltose aerobic (2 ug RNA)	GSE4877
GSM109609$ mid log LB 2 ug RNA aerobic	GSE4877
GSM109609$ mid log M9 + 0.5% maltose aerobic (2 ug RNA)	GSE4877
GSM143457$ wt mid log reference RNA	GSE24408
GSM143457$ wt RNA (from vibrio in ileal loop fluid) 12h rabbit	GSE24408
GSM143458$ wt mid log in LB RNA (2 ug)	GSE24408
GSM143458$ wt RNA (from vibrio in ileal loop fluid) 12h rabbit	GSE24408
GSM143459$ wt mid log reference RNA	GSE24408
GSM143459$ wt RNA (from vibrio in ileal loop fluid) 12h rabbit	GSE24408
GSM143460$ wt mid log in LB RNA (2 ug)	GSE24408
GSM143460$ wt RNA (from vibrio in ileal loop fluid) 12h rabbit	GSE24408
GSM143461$ wt mid log in LB RNA (2 ug)	GSE24408
GSM143461$ wt RNA (from vibrio in ileal loop fluid) 12h rabbit	GSE24408
GSM143462$ wt mid log reference RNA	GSE24408

GSM143462$ wt RNA (from vibrio in ileal loop fluid) 12h rabbit	GSE24408
GSM143463$ wt mid log in LB RNA (2 ug)	GSE24408
GSM143463$ wt RNA (from vibrio in ileal loop fluid) 12h rabbit	GSE24408
GSM143464$ wt mid log reference RNA	GSE24408
GSM143464$ wt RNA (from vibrio in ileal loop fluid) 12h rabbit	GSE24408
GSM143465$ A1552 11hr 3	GSE6220
GSM143465$ RpoS 11hr 2	GSE6220
GSM143466$ A1552 11hr 3	GSE6220
GSM143466$ RpoS 11hr 2	GSE6220
GSM143467$ A1552 11hr 3	GSE6220
GSM143467$ RpoS 11hr 2	GSE6220
GSM143468$ A1552 11hr 3	GSE6220
GSM143468$ RpoS 11hr 2	GSE6220
GSM143469$ A1552 11hr 3	GSE6220
GSM143469$ HapR 11hr 1	GSE6220
GSM143470$ wt 11h in LB 37 C (2 ug RNA)	GSE6220
GSM143470$ rpoS mutant 11h in LB 37C (2 ugRNA)	GSE6220
GSM143471$ A1552 11hr 3	GSE6220
GSM143471$ HapR 11hr 1	GSE6220
GSM143472$ wt 11h in LB 37 C (2 ug RNA)	GSE6220
GSM143472$ rpoS mutant 11h in LB 37C (2 ugRNA)	GSE6220
GSM143473$ A1552 11hr 3	GSE6220
GSM143473$ HapR 11hr 1	GSE6220
GSM143474$ wt mid log in LB 37 C (2 ug RNA)	GSE6220
GSM143474$ wt 11h in LB 37C (2 ug RNA)	GSE6220
GSM143475$ wt 11h in LB 37 C (2ug RNA)	GSE6220
GSM143475$ hapR mutant 11h in LB 37 C (2ug RNA)	GSE6220
GSM143476$ wt 11h in LB 37 C (2 ug RNA)	GSE6220
GSM143476$ rpoS mutant 11h in LB 37C (2 ugRNA)	GSE6220
GSM143477$ wt mid log in LB 37 C (2 ug RNA)	GSE6220

GSM143477$ wt 11h in LB 37C (2 ug RNA)	GSE6220
GSM143478$ wt 11h in LB 37 C (2ug RNA)	GSE6220
GSM143478$ hapR mutant 11h in LB 37 C (2ug RNA)	GSE6220
GSM143479$ wt mid log in LB 37 C (2 ug RNA)	GSE6220
GSM143479$ wt 11h in LB 37C (2 ug RNA)	GSE6220
GSM143480$ wt 11h in LB 37 C (2ug RNA)	GSE6220
GSM143480$ hapR mutant 11h in LB 37 C (2ug RNA)	GSE6220
GSM143481$ wt 11h in LB 37 C (2 ug RNA)	GSE6220
GSM143481$ rpoS mutant 11h in LB 37C (2 ugRNA)	GSE6220
GSM143482$ wt mid log in LB 37 C (2 ug RNA)	GSE6220
GSM143482$ wt 11h in LB 37C (2 ug RNA)	GSE6220
GSM143483$ wt 11h in LB 37 C (2 ug RNA)	GSE6220
GSM143483$ rpoS mutant 11h in LB 37C (2 ugRNA)	GSE6220
GSM359157$ wt mid log ref for AKI (combined from several experiments) 2 ug	GSE14360
GSM359157$ 2 h AKI (2 ug)	GSE14360
GSM359158$ wt mid log ref for AKI (combined from several experiments) 2 ug	GSE14360
GSM359158$ 5h AKI (2 ug)	GSE14360
GSM359159$ wt mid log ref for AKI (combined from several experiments) 2 ug	GSE14360
GSM359159$ 1 h AKI (2 ug)	GSE14360
GSM359160$ wt mid log ref for AKI (combined from several experiments) 2 ug	GSE14360
GSM359160$ 9h AKI (2 ug)	GSE14360
GSM359161$ wt mid log ref for AKI (combined from several experiments) 2 ug	GSE14360
GSM359161$ 6h AKI (2 ug)	GSE14360

GSM359162$ wt mid log ref for AKI (combined from several experiments) 2 ug	GSE14360
GSM359162$ 3 h AKI (2 ug)	GSE14360
GSM359163$ wt mid log ref for AKI (combined from several experiments) 2 ug	GSE14360
GSM359163$ 8h AKI (2 ug)	GSE14360
GSM359164$ wt mid log ref for AKI (combined from several experiments) 2 ug	GSE14360
GSM359164$ 7h AKI (2 ug)	GSE14360
GSM359165$ wt mid log ref for AKI (combined from several experiments) 2 ug	GSE14360
GSM359165$ 4h AKI (2 ug)	GSE14360
GSM359166$ wt mid log ref for AKI (combined from several experiments) 2 ug	GSE14360
GSM359166$ 10h AKI (2 ug)	GSE14360
GSM601459$ wt mid log reference RNA/ rabbit RNA (1:2)	GSE24408
GSM601459$ wt RNA (from vibrio attached to epithelium) 8h rabbit	GSE24408
GSM601460$ wt mid log in LB RNA (2 ug)	GSE24408
GSM601460$ wt RNA (from vibrio attached to epithelium) 8h rabbit	GSE24408
GSM601461$ wt mid log reference RNA/ rabbit RNA (1:2)	GSE24408
GSM601461$ wt RNA (from vibrio attached to epithelium) 8h rabbit	GSE24408
GSM601462$ wt mid log reference RNA/ rabbit RNA (1:2)	GSE24408
GSM601462$ wt RNA (from vibrio attached to epithelium) 8h rabbit	GSE24408
GSM601463$ wt mid log in LB RNA (2 ug)	GSE24408
GSM601463$ wt RNA (from vibrio attached to epithelium) 8h rabbit	GSE24408

GSM601464$ wt mid log reference RNA/ rabbit RNA (1:2)	GSE24408
GSM601464$ wt RNA (from vibrio attached to epithelium) 8h rabbit	GSE24408
GSM601465$ wt mid log in LB RNA (2 ug)	GSE24408
GSM601465$ wt RNA (from vibrio attached to epithelium) 8h rabbit	GSE24408
GSM601466$ wt mid log in LB RNA (2 ug)	GSE24408
GSM601466$ wt RNA (from vibrio attached to epithelium) 8h rabbit	GSE24408
GSM601467$ wt mid log reference in LB 37 C (2 ug RNA)	GSE24408
GSM601467$ wt RNA (from vibrio attached to epothelium) 12h rabbit	GSE24408
GSM601468$ wt mid log reference RNA/ rabbit RNA (1:2.5)	GSE24408
GSM601468$ wt RNA (from vibrio attached to epithelium) 12h rabbit	GSE24408
GSM601469$ wt mid log reference RNA/ rabbit RNA (1:2.5)	GSE24408
GSM601469$ wt RNA (from vibrio attached to epithelium) 12h rabbit	GSE24408
GSM601470$ wt mid log in LB 2 ug RNA	GSE24408
GSM601470$ wt RNA (from vibrio attached to epothelium) 12h rabbit	GSE24408
GSM601471$ wt mid log in LB 2 ug RNA	GSE24408
GSM601471$ wt RNA (from vibrio attached to epothelium) 12h rabbit	GSE24408
GSM601472$ wt mid log in LB 2 ug RNA	GSE24408
GSM601472$ wt RNA (from vibrio attached to epothelium) 12h rabbit	GSE24408
GSM601473$ wt mid log reference RNA/ rabbit RNA (1:2.5)	GSE24408
GSM601473$ wt RNA (from vibrio attached to epithelium) 12h rabbit	GSE24408
GSM601474$ wt mid log reference RNA/ rabbit RNA (1:2.5)	GSE24408

GSM601474$ wt RNA (from vibrio attached to epothelium) 12h rabbit	GSE24408
GSM601475$ wt mid log reference RNA/ rabbit RNA (1:1.5) 2 ugRNA	GSE24408
GSM601475$ wt in fluid (2 ug RNA)	GSE24408
GSM601476$ wt mid log reference RNA/ rabbit RNA (1:1.5) 2 ugRNA	GSE24408
GSM601476$ wt in fluid (2 ug RNA)	GSE24408
GSM601477$ wt mid log reference RNA/ rabbit RNA (1:2)	GSE24408
GSM601477$ wt RNA (from vibrio in ileal loop fluid) 8h rabbit	GSE24408
GSM601478$ 2ug mid log reference	GSE24408
GSM601478$ 2ug vc RNA from 8 h rabbit loop	GSE24408
GSM601479$ wt mid log reference RNA/ rabbit RNA (1:2)	GSE24408
GSM601479$ wt RNA (from vibrio in ileal loop fluid) 8h rabbit	GSE24408
GSM601480$ wt mid log reference RNA/ rabbit RNA (1:2)	GSE24408
GSM601480$ wt RNA (from vibrio in ileal loop fluid) 8h rabbit	GSE24408
GSM601481$ 2ug mid log reference	GSE24408
GSM601481$ 2ug vc RNA from 8 h rabbit loop	GSE24408
GSM601482$ mid log reference LB 37 C 1.5 ug RNA	GSE24408
GSM601482$ 8h ileal loop 1.5 ug RNA	GSE24408
GSM601483$ wt mid log reference RNA/ rabbit RNA (1:1.5) 2 ugRNA	GSE24408
GSM601483$ wt in fluid (2 ug RNA)	GSE24408
GSM69289$ M9 + 0.5% lacate	GSE4388
GSM69289$ M9 + 0.5% lactate + 0.6mM chitohexaose	GSE4388
GSM69290$ M9 + 0.5 % lactate	GSE4388

GSM69290$ M9 + 0.5 % lactate + 0.6 mM chitosan dimer	GSE4388
GSM69291$ M9 + 0.5 % lactate	GSE4388
GSM69291$ M9 + 0.5 % lactate + 0.6 mM chitosan dimer	GSE4388
GSM69292$ M9 + 0.5% lacate	GSE4388
GSM69292$ M9 + 0.5% lactate + 0.6mM chitohexaose	GSE4388
GSM69293$ M9 + 0.5% lactate	GSE4388
GSM69293$ M9 + 0.5% lactate + 0.6 mM chitosan dimer	GSE4388
GSM69294$ M9 + 0.5% lacate	GSE4388
GSM69294$ M9 + 0.5% lactate + 0.6mM chitohexaose	GSE4388
GSM69295$ M9 + 0.5% lactate	GSE4388
GSM69295$ M9 + 0.5% lactate + 0.6 mM chitosan dimer	GSE4388
GSM69296$ M9 + 0.5% lacate	GSE4388
GSM69296$ M9 + 0.5% lactate + 0.6mM chitohexaose	GSE4388
GSM69297$ M9 + 0.5% lactate	GSE4388
GSM69297$ M9 + 0.5% lactate + 0.6 mM chitotetraose	GSE4388
GSM69298$ M9 + 0.5% lactate	GSE4388
GSM69298$ M9 + 0.5% lactate + 0.6 mM chitobiose	GSE4388
GSM69299$ M9 + lactate	GSE4388
GSM69299$ M9 + lactate + 0.6 mM chitotetraose	GSE4388
GSM69300$ M9 + lactate	GSE4388
GSM69300$ M9 + lactate + 0.6 mM chitotetraose	GSE4388
GSM69301$ M9 + 0.5% lactate	GSE4388
GSM69301$ M9 + 0.5% lactate + 0.6 mM chitopentaose	GSE4388
GSM69302$ M9 + 0.5 % lactate	GSE4388

GSM69302$ M9 + 0.5% lactate + 0.6 mM chitopentaose	GSE4388
GSM69303$ M9 + 0.5 % lactate	GSE4388
GSM69303$ M9 + 0.5% lactate + 0.6 mM chitopentaose	GSE4388
GSM69304$ M9 + 0.5% lactate	GSE4388
GSM69304$ M9 + 0.5% lactate + 0.6 mM chitopentaose	GSE4388
GSM69305$ M9 + 0.5% lactate	GSE4388
GSM69305$ M9 + 0.5% lactate + 0.6 mM chitobiose	GSE4388
GSM69306$ M9 + 0.5 % lactate	GSE4388
GSM69306$ M9 + 0.5 % lactate + 0.6 mM chitosan dimer	GSE4388
GSM69307$ M9 + 0.5% lactate	GSE4388
GSM69307$ M9 + 0.5% lactate + 0.6 mM chitobiose	GSE4388
GSM69308$ M9 + 0.5 % lactate	GSE4388
GSM69308$ M9 + 0.5 % lactate + 0.6 mM chitosan dimer	GSE4388
GSM69309$ M9 + 0.5% lactate	GSE4388
GSM69309$ M9 + 0.5% lactate + 0.6 mM chitotriose	GSE4388
GSM69310$ M9 + 0.5% lactate	GSE4388
GSM69310$ M9 + 0.5% lactate + 0.6 mM chitobiose	GSE4388
GSM69311$ M9 + 0.5% lactate	GSE4388
GSM69311$ M9 + 0.5% lactate + 0.6 mM chitotriose	GSE4388
GSM69312$ M9 + 0.5% lactate	GSE4388
GSM69312$ M9 + 0.5% lactate + 0.6 mM chitobiose	GSE4388
GSM69313$ M9 + 0.5% lactate	GSE4388
GSM69313$ M9 + 0.5% lactate + 0.6 mM GlcNAc	GSE4388

GSM69314$ M9 + 0.5% lactate	GSE4388
GSM69314$ M9 + 0.5% lactate + 0.6 mM chitotriose	GSE4388
GSM69315$ M9 + 0.5% lactate	GSE4388
GSM69315$ M9 + 0.5% lactate + 0.6 mM GlcNAc	GSE4388
GSM69316$ M9 + 0.5% lactate	GSE4388
GSM69316$ M9 + 0.5% lactate + 0.6 mM chitotriose	GSE4388
GSM69317$ M9 + 0.5% lactate	GSE4388
GSM69317$ M9 + 0.5% lactate + 0.6 mM chitotriose	GSE4388
GSM69318$ M9 + 0.5% lactate	GSE4388
GSM69318$ M9 + 0.5% lactate + 0.6 mM GlcNAc	GSE4388
GSM69319$ M9 + 0.5% lactate	GSE4388
GSM69319$ M9 + 0.5% lactate + 0.6 mM chitotriose	GSE4388
GSM69320$ M9 + 0.5% lactate	GSE4388
GSM69320$ M9 + 0.5% lactate + 0.6 mM GlcNAc	GSE4388
GSM69321$ M9 + 0.5% lactate	GSE4388
GSM69321$ M9 + 0.5% lactate + 0.6mM chitotetraose	GSE4388
GSM69322$ M9 + 0.5% lactate	GSE4388
GSM69322$ M9 + 0.5% lactate + 0.6 mM chitotetraose	GSE4388
GSM69323$ M9 + 0.5% lactate	GSE4388
GSM69323$ M9 + 0.5% lactate + 0.6 mM GlcNAc	GSE4388
GSM69324$ M9 + 0.5% lactate	GSE4388
GSM69324$ M9 + 0.5% lactate + 0.6 mM chitotetraose	GSE4388
GSM69325$ M9 + 0.5% lactate	GSE4388
GSM69325$ M9 + 0.5% lactate + 0.6 mM GlcNAc	GSE4388
GSM69326$ M9 + 0.5% lactate	GSE4388

GSM69326$ M9 + 0.5% lactate + 0.6 mM chitohexaose	GSE4388
GSM69327$ M9 + 0.5% lactate	GSE4388
GSM69327$ M9 + 0.5% lactate + 0.6 mM chitohexaose	GSE4388
GSM69328$ M9 + 0.5% lactate	GSE4388
GSM69328$ M9 + 0.5% lactate + 0.6 mM chitobiose	GSE4388
GSM69329$ M9 + 0.5% lactate	GSE4388
GSM69329$ M9 + 0.5% lactate + 0.6 mM chitohexaose	GSE4388
GSM69330$ M9 + 0.5% lactate	GSE4388
GSM69330$ M9 + 0.5% lactate + 0.6 mM chitohexaose	GSE4388
GSM69331$ M9 + 0.5% lactate	GSE4388
GSM69331$ M9 + 0.5% lactate + 0.6 mM chitosan dimer	GSE4388
GSM69332$ M9 + 0.5% lactate	GSE4388
GSM69332$ M9 + 0.5% lactate + 0.6 mM chitosan dimer	GSE4388
GSM69333$ M9 + 0.5% lactate	GSE4388
GSM69333$ M9 + 0.5% lactate + 0.6 mM chitopentaose	GSE4388
GSM69334$ M9 + 0.5% lactate	GSE4388
GSM69334$ M9 + 0.5% lactate + 0.6 mM chitopentaose	GSE4388
GSM69335$ M9 + lactate	GSE4388
GSM69335$ M9 + lactate + 0.6 mM chitotetraose	GSE4388
GSM69336$ M9 + 0.5% lactate	GSE4388
GSM69336$ M9 + 0.5% lactate + 0.6 mM chitobiose	GSE4388
GSM69337$ M9 + 0.5% lactate	GSE4388
GSM69337$ M9 + 0.5% lactate + 0.6 mM chitobiose	GSE4388
GSM69338$ M9 + 0.5% lactate	GSE4388

GSM69338$ M9 + 0.5% lactate + 0.6mM chitotetraose	GSE4388
GSM69339$ M9 + 0.5% lactate	GSE4388
GSM69339$ M9 + 0.5% lactate + 0.6 mM chitopentaose	GSE4388
GSM69340$ M9 + 0.5% lactate	GSE4388
GSM69340$ M9 + 0.5% lactate + 0.6mM chitotriose	GSE4388
GSM69341$ M9 + 0.5% lactate	GSE4388
GSM69341$ M9 + 0.5% lactate + 0.6 mM chitopentaose	GSE4388
GSM69342$ M9 + 0.5% lactate	GSE4388
GSM69342$ M9 + 0.5% lactate + 0.6 mM GlcNAc	GSE4388
GSM69343$ M9 + 0.5% lactate	GSE4388
GSM69343$ M9 + 0.5% lactate + 0.6mM chitotriose	GSE4388
GSM69344$ M9 + 0.5% lactate	GSE4388
GSM69344$ M9 + 0.5% lactate + 0.6 mM GlcNAc	GSE4388
GSM69345$ planktonic bacteria	GSE4388
GSM69345$ crab attached bacteria	GSE4388
GSM69346$ Planktonic bacteria	GSE4388
GSM69346$ crab attached bacteria	GSE4388
GSM69347$ Planktoninc bacteria	GSE4388
GSM69347$ Crab attached bacteria	GSE4388
GSM69348$ Planktoninc bacteria	GSE4388
GSM69348$ Crab attached bacteria	GSE4388
GSM69349$ planktonic bacteria	GSE4388
GSM69349$ crab attached bacteria	GSE4388
GSM69350$ planktonic bacteria	GSE4388
GSM69350$ crab attached bacteria	GSE4388
GSM69351$ planktonic bacteria	GSE4388

GSM69351$ crab attached bacteria	GSE4388
GSM69352$ planktonic bacteria	GSE4388
GSM69352$ crab attached bacteria	GSE4388
GSM69353$ planktonic bacteria	GSE4388
GSM69353$ crab attached bacteria	GSE4388
GSM69354$ planktonic bacteria	GSE4388
GSM69354$ crab attached bacteria	GSE4388
GSM69355$ VCXB21: M9 + 0.5% lactate + 0.6 mM chitobiose	GSE4388
GSM69355$ VCXB21pDel: M9 + 0.5% lactate + 0.6 mM chitobiose	GSE4388
GSM69356$ VCXB21: M9 + 0.5% lactate + 0.6 mM chitobiose	GSE4388
GSM69356$ VCXB21pDel: M9 + 0.5% lactate + 0.6 mM chitobiose	GSE4388
GSM69357$ VCXB21: M9 + 0.5% lactate + 0.6 mM chitobiose	GSE4388
GSM69357$ VCXB21pDel: M9 + 0.5% lactate + 0.6 mM chitobiose	GSE4388
GSM69358$ VCXB21: M9 + 0.5% lactate + 0.6 mM chitobiose	GSE4388
GSM69358$ VCXB21pDel: M9 + 0.5% lacate + 0.6 mM chitobiose	GSE4388
GSM69361$ 1.5 ug Nadia exponential cDNA	GSE4877
GSM69361$ 1.5 ug O1 Inaba cDNA	GSE4877
GSM69362$ 1.5 ug Nadia exponential cDNA	GSE4877
GSM69362$ 1.5 ug O1 Inaba cDNA	GSE4877
GSM69363$ 1.5 ug Nadia exponential cDNA	GSE4877
GSM69363$ 1.5 ug O1 Inaba cDNA	GSE4877
GSM69364$ 2.0 ug Nadia exponential cDNA	GSE4877
GSM69364$ 2.0 ug O1 Inaba stationary cDNA	GSE4877
GSM69365$ 1.0 ug Nadia exponential cDNA	GSE4877
GSM69365$ 1.0 ug O1 Inaba cDNA	GSE4877

GSM69366$ 2.0 ug Nadia exponential cDNA	GSE4877
GSM69366$ 2.0 ug O1 Inaba stationary cDNA	GSE4877
GSM69367$ 1.0 ug Nadia exponential cDNA	GSE4877
GSM69367$ 1.0 ug O1 Inaba cDNA	GSE4877
GSM69368$ 2.0 ug Nadia exponential cDNA	GSE4877
GSM69368$ 2.0 ug O1 Inaba stationary cDNA	GSE4877
GSM69369$ 1.5 ug Nadia exponential cDNA	GSE4877
GSM69369$ 1.5 ug O1 Inaba cDNA	GSE4877
GSM69370$ 1.0 ug Nadia exponential cDNA	GSE4877
GSM69370$ 1.0 ug O1 Inaba cDNA	GSE4877
GSM69371$ 2.0 ug Nadia exponential cDNA	GSE4877
GSM69371$ 2.0 ug O1 Inaba cDNA	GSE4877
GSM69372$ 1.0 ug Nadia exponential cDNA	GSE4877
GSM69372$ 1.0 ug O1 Inaba cDNA	GSE4877
GSM69373$ 2.0 ug Nadia exponential cDNA	GSE4877
GSM69373$ 2.0 ug O1 Inaba stationary cDNA	GSE4877
GSM69374$ 2.0 ug Nadia exponential cDNA	GSE4877
GSM69374$ 2.0 ug O1 Inaba cDNA	GSE4877
GSM69375$ 2.0 ug Nadia exponential cDNA	GSE4877
GSM69375$ 2.0 ug O1 Inaba cDNA	GSE4877
GSM69376$ 2.0 ug Nadia exponential cDNA	GSE4877
GSM69376$ 2.0 ug O1 Inaba cDNA	GSE4877
GSM82276$ wt induced (2 ug RNA)	GSE3576
GSM82276$ hapR mutant induced (2 ug RNA)	GSE3576
GSM82277$ wt induced (2 ug RNA)	GSE3576
GSM82277$ hapR mutant induced (2 ug RNA)	GSE3576
GSM82278$ wt induced (3 ug RNA)	GSE3576
GSM82278$ hapR mutant induced (3ug RNA)	GSE3576
GSM82279$ wt induced (3 ug RNA)	GSE3576

GSM82279$ hapR mutant induced (3ug RNA)	GSE3576
GSM82280$ A1552tfoX/pBAD	GSE3577
GSM82280$ A1552tfoX/pBAD-tfoX	GSE3577
GSM82281$ A1552tfoX/pBAD	GSE3577
GSM82281$ A1552tfoX/pBAD-tfoX	GSE3577
GSM82282$ A1552tfoX/pBAD	GSE3577
GSM82282$ A1552tfoX/pBAD-tfoX	GSE3577
GSM82283$ A1552tfoX/pBAD	GSE3577
GSM82283$ A1552tfoX/pBAD-tfoX	GSE3577
GSM936374$ 2.0 ug starting cDNA	GSE38179
GSM936374$ 2.0 ug slowly frozen cDNA	GSE38179
GSM936375$ 2.0 ug starting cDNA	GSE38179
GSM936375$ 2.0 ug slowly frozen cDNA	GSE38179
GSM936376$ 2.0 ug starting cDNA	GSE38179
GSM936376$ 2.0 ug slowly frozen cDNA	GSE38179
GSM936377$ 2.0 ug starting cDNA	GSE38179
GSM936377$ 2.0 ug slowly frozen cDNA	GSE38179
GSM148648$Bacteria.was.grown.in.the.presence.of.0.4..bile.for.5.5.hours	GSE6468
GSM148648$Bacteria.was.grown.in.the.abscence.of.bile.for.5.5.hours	GSE6468
GSM148656$Bacteria.was.grown.in.the.presence.of.0.4..bile.for.5.5.hours	GSE6468
GSM148656$Bacteria.was.grown.in.the.abscence.of.bile.for.5.5.hours	GSE6468
GSM265512$Bacteria.was.grown.in.the.abscence.of.bile.for.2.hours	GSE6468
GSM265512$Bacteria.was.grown.in.the.presence.of.0.02..bile.for.2.hours	GSE6468
GSM265529$Bacteria.was.grown.in.the.abscence.of.bile.for.2.hours	GSE6468
GSM265529$Bacteria.was.grown.in.the.presence.of.0.02..bile.for.2.hours	GSE6468

GSM265541$Bacteria.was.grown.in.the.abscence.of.bile.for.2.hours	GSE6468
GSM265541$Bacteria.was.grown.in.the.presence.of.0.02..bile.for.2.hours	GSE6468
GSM265542$Bacteria.was.grown.in.the.abscence.of.bile.for.2.hours	GSE6468
GSM265542$Bacteria.was.grown.in.the.presence.of.0.4..bile.for.2.hours	GSE6468
GSM265543$Bacteria.was.grown.in.the.abscence.of.bile.for.2.hours	GSE6468
GSM265543$Bacteria.was.grown.in.the.presence.of.0.4..bile.for.2.hours	GSE6468
GSM265544$Bacteria.was.grown.in.the.abscence.of.bile.for.2.hours	GSE6468
GSM265544$Bacteria.was.grown.in.the.presence.of.0.4..bile.for.2.hours	GSE6468
GSM265545$Bacteria.was.grown.in.the.abscence.of.bile.for.4.hours	GSE6468
GSM265545$Bacteria.was.grown.in.the.presence.of.0.4..bile.for.4.hours	GSE6468
GSM265547$Bacteria.was.grown.in.the.abscence.of.bile.for.4.hours	GSE6468
GSM265547$Bacteria.was.grown.in.the.presence.of.0.4..bile.for.4.hours	GSE6468
GSM265548$Bacteria.was.grown.in.the.abscence.of.bile.for.4.hours	GSE6468
GSM265548$Bacteria.was.grown.in.the.presence.of.0.4..bile.for.4.hours	GSE6468
GSM45609$V..cholerae.recovered.from.human.stool	GSE2775
GSM45609$V..cholerae.recovered.from.human.vomitus	GSE2775
GSM148647$Bacteria.was.grown.in.the.presence.of.0.4..bile.for.5.5.hours	GSE6468
GSM148647$Bacteria.was.grown.in.the.abscence.of.bile.for.5.5.hours	GSE6468
