# Supplementary material for: Cross-Platform Transcriptomic Data Integration, Profiling, and Mining in Vibrio cholerae
Source: Microbiol Spectr. 2023 May 16;11(3):e05369-22. doi: 10.1128/spectrum.05369-22 (PMC10269641; doi:10.1128/spectrum.05369-22)
Supplement: Supplemental file 3 — Supplemental material. Download spectrum.05369-22-s0003.pdf, PDF file, 0.1 MB [file spectrum.05369-22-s0003.pdf]

Group3			
Sample ID	PCA.1	PCA.2	Distance
SRR5110873	-2.03137	-3.14043	5.064991
SRR5110870	-1.93232	-2.85108	5.370315
SRR10905381	-2.8025	-1.90256	6.146419
SRR10905382	-2.72769	-1.78691	6.268957
SRR9289627	-0.82992	-2.11584	6.434305
SRR3586860	-1.01565	-1.73904	6.714039
SRR3586871	-1.15105	-1.53818	6.85706
SRR5938305	-0.88237	-1.61967	6.87325
SRR3586863	-1.41602	-1.40757	6.90048
SRR10387917	0.063882	-1.96169	6.972071
SRR5938201	-0.7041	-1.56151	6.993806
SRR5938306	-0.96439	-1.44195	7.01034
SRR10387915	-1.02758	-1.357	7.068807
SRR5110872	-1.13418	-1.2118	7.171709
SRR7298349	-1.77885	-1.02075	7.182357
SRR10387916	-0.7446	-1.31168	7.210278
SRR3586876	-0.46267	-1.35996	7.273953
SRR7298350	-1.9582	-0.79076	7.369919
SRR8294795	-0.4088	-1.26419	7.383304
SRR3586879	-0.38333	-1.24286	7.41311
SRR5110871	-1.16542	-0.93244	7.428086
SRR8294796	-0.37222	-1.18951	7.466396
SRR8294799	-0.33202	-1.19575	7.477001
SRR5110874	-0.72453	-1.01768	7.491739
SRR5112947	-0.48563	-1.03457	7.564242
SRR10905370	-1.41011	-0.63162	7.648923
SRR10905369	-1.59153	-0.55327	7.680282
SRR8447494	-0.49495	-0.85983	7.722444
SRR7298351	-2.02595	-0.40637	7.73521
SRR8447495	-0.14268	-0.97878	7.752827
SRR442064	-0.99593	-0.54466	7.848926
SRR5938200	0.049696	-0.91551	7.892134
SRR5938168	0.020209	-0.89978	7.893537
SRR8447493	-1.27219	-0.3927	7.91566
SRR442059	0.207948	-0.88909	7.985851
SRR5938202	0.243988	-0.89633	7.995673
SRR5938307	0.019788	-0.74223	8.035785
SRR7298354	-0.92502	-0.36625	8.040469
SRR5112948	-0.76477	-0.37788	8.079911
SRR8294800	0.040288	-0.67865	8.102066
SRR1636728	-0.89483	-0.30613	8.106947
SRR8447498	0.058002	-0.65908	8.127294
SRR442060	0.115592	-0.65526	8.155307
SRR442062	-0.45999	-0.40537	8.158141
SRR8447497	-0.18102	-0.48952	8.184732
SRR442063	-0.39006	-0.40278	8.185873
SRR7298353	-1.32968	-0.08957	8.193598
SRR5938204	0.254683	-0.65991	8.211785
SRR5938304	0.146738	-0.60495	8.214114
SRR7298352	-1.18809	-0.08006	8.239407
SRR8294797	0.049251	-0.51692	8.252649
SRR1636727	0.035933	-0.42908	8.327128
SRR8447496	-0.78421	-0.09776	8.339318
SRR8447501	0.672822	-0.71223	8.359663
SRR1636729	-0.02634	-0.3512	8.373024
SRR8284475	2.01064	-1.60639	8.381974

Group4			
Sample ID	PCA.1	PCA.2	Distance
SRR10387918	139.1362	-43.0344	44.93918
SRR9313021	132.3678	-49.4646	51.64713
SRR10387921	141.9342	-57.2337	59.25971
SRR13362017	161.849	63.4548	66.06797
SRR10387922	127.3426	-63.4827	66.21419
SRR9313022	145.035	-67.0805	69.33776
SRR13362019	179.8471	58.52568	70.49098
SRR1521334	72.78452	-26.983	71.21219
SRR8284470	66.55782	7.948744	71.58128
SRR8284473	66.49278	12.11056	72.11752
SRR10905391	170.5143	-62.7961	72.44869
SRR13362016	178.9631	62.01489	72.82159
SRR13362018	181.4938	62.67606	74.81488
SRR8284471	63.17496	7.808287	74.94131
SRR10905393	174.1925	-64.1562	75.36706
SRR10905392	172.7508	-65.1709	75.58191
SRR13362022	97.0824	67.84831	77.55942
SRR1521335	65.59023	-31.768	79.74197
SRR10905385	173.0156	-69.8566	79.88465
SRR10905383	170.9455	-70.878	79.92491
SRR10905386	169.2495	-71.7188	80.0111
SRR13362020	72.36922	55.36747	84.56982
SRR1521336	55.2391	-17.8263	84.96161
SRR1521337	57.99717	-29.102	85.68501
SRR10905376	71.67484	-61.0399	91.34125
SRR12867128	78.39731	-69.6145	93.01043
SRR12867121	86.70616	-76.9825	94.01813
SRR10905378	60.11489	-51.4445	94.29743
SRR13362023	68.62742	66.44728	94.67941
SRR12867130	78.90039	-74.313	96.36033
SRR10905377	63.95482	-60.3915	96.66369
SRR10905379	57.30862	-53.0476	97.51879
SRR10905389	178.1994	-86.9457	97.55408
SRR10905380	54.03497	-48.3452	97.74264
SRR12867127	74.41004	-73.1059	98.24802
SRR2302171	63.07263	-67.5535	102.0709
SRR6334014	241.1969	12.54404	103.863
SRR12867129	56.10944	-66.8161	106.8031
SRR13362021	45.3752	60.5361	109.5317
SRR2648182	30.99036	-33.795	112.6901
SRR12867122	88.23727	-99.6574	113.0306
SRR6334024	250.2547	14.68306	113.0989
SRR6334013	252.3114	-15.9465	115.8108
SRR6334012	252.0029	-21.8358	116.5606
SRR2302173	22.23901	-20.2978	117.7518
SRR6334011	256.214	6.663469	118.4283
SRR6334017	256.4273	-3.60659	118.6725
SRR6334018	256.7031	-16.4408	120.2263
SRR2302172	19.47116	-34.171	123.7794
SRR6334015	246.9068	-59.883	125.3076
SRR6334016	263.5865	10.27381	125.984
SRR6334023	265.6401	4.765224	127.7906
SRR6334025	266.3729	-5.5106	128.7036
SRR6334021	264.6514	-26.8523	129.9863
SRR6334022	267.7409	-12.7694	130.6834
SRR10905388	172.8029	-125.714	132.293

SRR8284472	2.290812	-1.84757	8.38587	SRR6334020	270.6646	22.37244	134.3535
SRR3586855	0.386061	-0.52032	8.395331	SRR10905387	173.6979	-127.807	134.5489
SRR8447500	0.428645	-0.52786	8.407813	SRR6334019	273.4181	-18.9455	137.1278
SRR1636730	-1.53769	0.193041	8.419423	SRR2648183	4.091103	-47.1376	142.4902
SRR8447499	0.398626	-0.49434	8.424186	SRR7298348	77.23597	134.5698	145.8855
SRR8284474	2.01625	-1.48948	8.475353	SRR7298346	69.17486	134.3817	149.2496
SRR5938203	0.016337	-0.25778	8.475717	SRR7298347	69.37132	138.1022	152.4737
SRR442061	0.645491	-0.52396	8.511785	SRR7298337	58.41313	137.0886	156.8269
SRR6987764	-1.36962	0.29335	8.555331	SRR7298338	56.45706	137.0965	157.8339
SRR3586853	0.415799	-0.3357	8.573825	SRR7298345	36.29895	123.821	158.7039
SRR6987762	-1.56618	0.362202	8.578462	SRR7298339	53.89469	136.6304	158.7752
SRR11546824	0.680539	-0.41682	8.622772	SRR7298343	36.76721	124.4765	158.9098
SRR11546823	0.987792	-0.55684	8.650696	SRR7298344	32.92674	127.6957	163.8394
SRR11546822	0.682588	-0.2582	8.763934	SRR7298334	17.0019	119.5818	168.7127
ERR5101713	-2.84907	0.783119	8.820753	SRR7298332	21.55194	125.2916	169.5916
ERR5101714	-2.75026	0.789015	8.83318	SRR7298333	23.05559	127.0413	169.8488
ERR5101712	-2.78909	0.852389	8.893709	SRR7298336	16.90348	122.4066	170.7651
SRR10905371	0.411739	0.041209	8.911524	SRR7298335	14.61095	120.1461	170.8242
SRR10905372	0.421679	0.107674	8.975912	SRR7298340	16.1567	121.9587	170.9801
SRR2319533	0.63072	0.156207	9.110067	SRR7298341	13.93413	120.6488	171.6606
SRR7178742	0.935964	0.002415	9.112956	SRR7298330	17.81156	125.0809	172.0279
SRR2319535	0.689549	0.184797	9.161746	SRR7298328	15.31938	125.2089	173.8673
SRR11546818	1.851016	-0.48252	9.178393	SRR7298329	15.1735	125.9106	174.4682
SRR2319530	0.532469	0.295498	9.193016	SRR7298331	19.19034	130.9461	175.3393
SRR2319532	0.580991	0.272743	9.193259	SRR7298342	10.15217	123.7668	176.5489
SRR11546817	1.838471	-0.44925	9.198611	SRR7298325	7.705653	121.8722	177.0374
SRR11546816	1.637632	-0.26949	9.23643	SRR7298327	4.12411	118.3295	177.3414
SRR7178741	0.957506	0.156887	9.259338	SRR7298326	4.593582	123.3213	180.3109
SRR2319534	0.74129	0.283291	9.272974	SRR9853387	-59.2399	-37.3093	200.9811
SRR7178745	0.395865	0.476	9.300275	SRR9853386	-62.5404	-28.6374	202.7335
SRR7178740	0.815121	0.281088	9.304091	SRR9853388	-61.7986	-36.1253	203.2666
ERR5101705	-3.54411	1.294178	9.316928	SRR2648177	-59.6521	-54.433	205.4062
SRR7178748	1.209909	0.12637	9.353747	SRR11194727	-67.4886	-18.6443	206.3943
SRR7178743	0.234275	0.606155	9.355664	SRR2648176	-60.2793	-57.1988	206.7827
SRR6987757	0.757241	0.372801	9.360269	SRR9853385	-70.2378	-32.0808	210.8737
SRR7178744	0.34256	0.591675	9.384786	SRR9853383	-69.6916	-35.717	210.9524
SRR7178739	1.368309	0.078339	9.391098	SRR11091461	-69.5154	-48.126	213.3425
SRR3371169	2.000518	-0.3219	9.39565	SRR5519753	-69.9341	-48.8443	213.9187
ERR5101704	-3.61592	1.374968	9.399203	SRR11091463	-71.4371	-46.4097	214.8187
ERR5101706	-3.68376	1.401651	9.427788	SRR11091464	-73.1298	-39.7187	215.0745
SRR3371170	2.212588	-0.40614	9.450769	SRR5519754	-70.8252	-50.7306	215.2378
SRR6987763	1.420333	0.126599	9.459073	SRR11091459	-73.7315	-39.4614	215.6154
SRR1382207	-3.04989	1.445278	9.472563	SRR11091462	-68.1557	-61.928	215.6941
SRR7178747	1.197454	0.269861	9.472969	SRR11091460	-70.346	-59.0034	216.9484
SRR7178746	1.088793	0.338038	9.480951	SRR2648178	-74.3273	-44.3113	217.1799
SRR3371168	2.098813	-0.25204	9.50932	SRR2648179	-75.3162	-41.1094	217.4907
SRR7178738	1.313222	0.271191	9.530658	SRR9853384	-77.0076	-35.4447	218.1082
SRR7178737	1.132945	0.373632	9.533191	SRR5519749	-74.3764	-48.5481	218.1682
ERR5101711	-3.05428	1.672289	9.699285	SRR5519751	-75.5343	-45.0054	218.5073
ERR5101709	-3.21496	1.714731	9.737584	SRR5519752	-76.6322	-45.8057	219.752
SRR3371166	1.048958	0.651846	9.740566	SRR5519750	-79.3931	-45.5784	222.3993
SRR7178730	2.385612	-0.17082	9.74285	SRR1636724	-82.079	-33.5364	222.7952
SRR3371165	1.064074	0.648899	9.744833	SRR11194734	-87.7351	-8.64455	225.8627
SRR6987761	1.586677	0.392886	9.772947	SRR12867134	-86.538	-35.957	227.5885
SRR3274038	2.097096	0.072474	9.775283	SRR12867135	-87.1828	-40.095	228.9469
ERR5101710	-3.10116	1.760009	9.785486	SRR11194672	-91.7173	-16.9272	230.3689
SRR10480747	-3.48458	1.767347	9.789241	SRR12867133	-90.7551	-26.5196	230.395
SRR7178729	2.432496	-0.14107	9.794615	SRR12867131	-91.3731	-23.1936	230.6229

SRR3274039	2.062718	0.125546	9.800123	SRR12867132	-90.0412	-34.4449	230.8008
SRR3371160	2.256686	-0.00714	9.80045	SRR12867136	-90.7475	-29.7197	230.8039
SRR10480746	-3.33674	1.781134	9.802629	SRR1636726	-91.0989	-30.5946	231.2733
SRR3274040	2.303586	-0.03364	9.805918	SRR11194677	-99.0962	-4.75431	237.0712
SRR3371167	1.157684	0.67107	9.807564	SRR11194721	-99.8192	-8.34089	237.9211
SRR10480745	-3.50977	1.792513	9.814695	SRR11194711	-100.24	7.456055	238.1872
SRR10985180	-3.50977	1.792513	9.814695	SRR2302174	-101.861	-23.2411	241.056
SRR7178734	2.373459	-0.05269	9.831162	SRR2302175	-104.032	-19.1804	242.8296
SRR7178728	2.335343	-0.02077	9.83484	SRR11194689	-105.685	0.016271	243.5743
SRR10480743	-3.6399	1.815376	9.840076	SRR5688349	-109.538	-26.2379	249.0134
SRR10985178	-3.6399	1.815376	9.840076	SRR5688347	-110.549	-27.9039	250.2109
SRR10480748	-3.46428	1.819945	9.84165	SRR2302176	-111.074	-24.8824	250.3916
SRR3371164	2.433661	-0.07848	9.845726	SRR5688346	-110.946	-26.5275	250.4451
SRR10480744	-3.58331	1.83337	9.85676	SRR11194735	-121.275	-23.0128	260.3503
SRR10985179	-3.58331	1.83337	9.85676	SRR11194728	-127.084	-15.4026	265.5292
SRR3371163	2.392123	-0.00919	9.877364	SRR11148749	-129.829	17.63206	268.1731
SRR10985183	-3.57818	1.854831	9.878114	SRR11148751	-131.927	17.65626	270.2693
SRR3274037	2.737795	-0.26473	9.881582	SRR11148752	-132.581	19.38178	271.0282
SRR7178733	2.130913	0.184776	9.887293	SRR11194722	-133.567	2.010925	271.4489
SRR10985182	-3.54256	1.872178	9.894824	SRR11148748	-133.412	18.75573	271.8184
SRR10985181	-3.5978	1.874031	9.897714	SRR11194695	-134.128	-7.6536	272.1768
SRR3274035	2.575765	-0.10039	9.912845	SRR11148747	-134.065	19.00283	272.4851
SRR7178736	2.62229	-0.10547	9.936838	SRR11194678	-134.734	-7.56994	272.78
SRR7178725	2.469756	0.009968	9.938437	SRR11148750	-134.498	20.32873	273.0039
SRR7178727	2.590846	-0.07459	9.942528	SRR11194705	-135.212	-17.8041	273.8031
SRR3274036	2.573309	-0.04294	9.957336	SRR8316516	-136.604	6.205842	274.5205
SRR7178735	2.599942	-0.06109	9.95878	SRR1636723	-136.304	-23.088	275.3207
SRR3371162	2.331627	0.1418	9.965407	SRR8316520	-137.768	2.254176	275.6499
ERR5101707	-2.73636	1.936581	9.979018	SRR11194733	-138.186	1.946566	276.0677
SRR7178732	2.182209	0.275217	9.990974	SRR11194716	-138.67	-5.00348	276.6374
SRR7178731	1.97731	0.40923	9.991099	SRR8316512	-140.097	4.22655	277.9889
SRR3371159	2.296575	0.233629	10.02085	SRR8316522	-140.53	0.816614	278.4144
ERR5101708	-2.59459	1.988584	10.04107	SRR8316513	-140.78	8.824374	278.7486
SRR7178726	2.681522	-0.0113	10.04767	SRR11194667	-141.462	-2.86148	279.3842
SRR9975254	-0.05616	1.525242	10.11012	SRR11194668	-141.597	3.847851	279.4859
SRR9975249	1.732813	0.736833	10.14353	SRR8316531	-141.624	1.471796	279.5066
SRR9975251	1.680762	0.774764	10.15022	SRR8316529	-141.66	1.258998	279.5425
SRR9975250	1.790625	0.724673	10.16235	SRR11194710	-141.469	-9.10905	279.5669
SRR9975253	-0.01026	1.618242	10.21305	SRR1636725	-141.925	-19.421	280.6171
SRR10985174	1.351959	1.07309	10.25385	SRR8316517	-142.806	5.331346	280.7091
SRR9975252	0.100268	1.639128	10.26974	SRR8316523	-142.933	5.874022	280.8431
SRR10985173	1.335831	1.150649	10.31532	SRR11194701	-143.51	5.083472	281.4106
SRR10985177	1.706955	0.951904	10.31701	SRR8316515	-143.635	2.163732	281.5169
SRR10985172	1.326488	1.20233	10.35704	SRR11194690	-144.066	-9.16856	282.1652
SRR10985175	1.782883	0.986978	10.3851	SRR8316521	-144.265	5.227482	282.1667
SRR3331928	0.222297	1.720965	10.38849	SRR8316519	-145.021	3.492345	282.9075
SRR3331926	0.281264	1.710857	10.39965	SRR8316530	-145.463	-0.31409	283.3539
SRR10985166	1.330609	1.261085	10.41127	SRR8316525	-146.632	1.814236	284.5142
SRR3331927	0.205314	1.77395	10.43234	SRR8316527	-147.394	4.488344	285.2883
SRR10985167	1.375481	1.267243	10.43715	SRR8316514	-148.3	0.557315	286.185
SRR10985168	1.372077	1.282155	10.44887	SRR8316526	-149.553	1.7803	287.4347
SRR10985176	1.860763	1.031587	10.46264	SRR8316518	-150.272	1.627092	288.1543
SRR3331925	0.359568	1.77196	10.48456	SRR8316528	-151.911	-0.10944	289.7996
SRR10985170	1.791719	1.158008	10.53814	SRR8316524	-155.082	2.020885	292.9638
SRR10985171	1.833995	1.18868	10.58564	SRR11194673	-157.493	-5.82979	295.4757
SRR10985169	1.805069	1.270199	10.64251				
SRR3331923	1.378859	1.58594	10.72326				
SRR3331922	1.855396	1.350022	10.73674				

SRR3331921	1.828873	1.404295	10.77128
SRR3331924	1.309553	1.717864	10.81134
